# Radiomics nomogram for preoperative differentiation of early-stage serous borderline ovarian tumors and serous malignant ovarian tumors

**DOI:** 10.3389/fonc.2023.1269589

**Published:** 2024-01-15

**Authors:** Xinping Yu, Yuwei Zou, Lei Wang, Hongjuan Yang, Jinwen Jiao, Haiyang Yu, Shuai Zhang

**Affiliations:** ^1^ Department of Gynecology, The Affiliated Hospital of Qingdao University, Qingdao, China; ^2^ Department of Pathology, The Affiliated Hospital of Qingdao University, Qingdao, China; ^3^ Department of Radiology, The Affiliated Hospital of Qingdao University, Qingdao, China

**Keywords:** ovarian tumors, multidetector computed tomography, radiomics, nomogram, preoperative differentiation

## Abstract

**Objectives:**

This study aimed to construct a radiomics nomogram and validate its performance in the preoperative differentiation between early-stage (I and II) serous borderline ovarian tumors (SBOTs) and serous malignant ovarian tumors (SMOTs).

**Methods:**

Data were collected from 80 patients with early-stage SBOTs and 102 with early-stage SMOTs (training set: *n* = 127; validation set: *n* = 55). Univariate and multivariate analyses were performed to identify the independent clinicoradiological factors. A radiomics signature model was constructed using radiomics features extracted from multidetector computed tomography images of the venous phase, in which the least absolute shrinkage and selection operator regression was employed to lessen the dimensionality of the data and choose the radiomics features. A nomogram model was established by combining independent clinicoradiological factors with the radiomics signature. The performance of nomogram calibration, discrimination, and clinical usefulness was evaluated using training and validation sets.

**Results:**

In terms of clinicoradiological characteristics, age (*p* = 0.001), the diameter of the solid component (*p* = 0.009), and human epididymis protein 4 level (*p* < 0.001) were identified as the independent risk factors of SMOT, for which the area under the curves (AUCs) were calculated to be 0.850 and 0.836 in the training and validation sets, respectively. Nine features were finally selected to construct the radiomics signature model, which exhibited AUCs of 0.879 and 0.826 for the training and validation sets, respectively. The nomogram model demonstrated considerable calibration and discrimination with AUCs of 0.940 and 0.909 for the training and validation sets, respectively. The nomogram model displayed more prominent clinical usefulness than the clinicoradiological and radiomics signature models according to the decision curve analysis.

**Conclusions:**

The nomogram model can be employed as an individualized preoperative non-invasive tool for differentiating early-stage SBOTs from SMOTs.

## Introduction

Epithelial ovarian tumors account for 60% of all ovarian tumors and are common in women all over the world (1). Ovarian epithelial tumors can be morphologically categorized into three types as follows: benign, borderline, and malignant (2), with borderline tumors accounting for 10%–15% of all primary ovarian tumors (3, 4). Typically, compared with serous malignant ovarian tumors (SMOTs), serous borderline ovarian tumors (SBOTs) have a better prognosis, implying the vitality of early diagnosis and treatment.

Over the past decade, increasing efforts have been made to differentiate between borderline ovarian tumors (BOTs) and malignant ovarian tumors (MOTs) to modify the differential diagnosis and allow for more timely and effective treatments. Nougaret et al. (5) demonstrated the efficient differentiation of SBOTs from low-grade SMOTs using multidetector computed tomography (MDCT) features, such as peritoneal disease patterns and solid tumor volumes. Furthermore, Grabowska-Derlatka et al. (6) indicated that the morphological assessment of tumor vascularity in MDCT is an efficient approach to distinguish BOTs from MOTs. Previous studies demonstrated that BOTs can be distinguished from MOTs by analyzing the apparent diffusion coefficient of a solid tumor component using magnetic resonance imaging (MRI) (7, 8). Differentiating BOTs from MOTs based on imaging alone is generally difficult; thus, some studies have attempted to predict BOTs using preoperative imaging and tumor biomarkers. A previous study validated the superior performance of combining MRI with serum cancer antigen 125 (CA125) for differentiating between BOTs and MOTs (9). In our previous study, age, vascular abnormalities, and largest solid portion were independent factors distinguishing SBOTs from SMOTs (10).

Radiomics is an emerging translational research field that aims to extract high-dimensional quantitative features from clinical images and provide useful information (11). This approach has been adopted in a variety of applications, including the prediction of treatment response and outcome, tumor staging, tissue identification, and cancer genetics assessment (12, 13). SBOTs and SMOTs share similar clinical symptoms and imaging features in their early stages, making differentiation between these two entities difficult. We developed a nomogram combining clinical characteristics, conventional imaging characteristics, and MDCT radiomics features for preoperative differentiation between early-stage SBOTs and SMOTs. As far as we know, it is the first nomogram model established for preoperative differentiation between early-stage SBOTs and SMOTs.

## Materials and methods

### Patients

**Figure 1:**
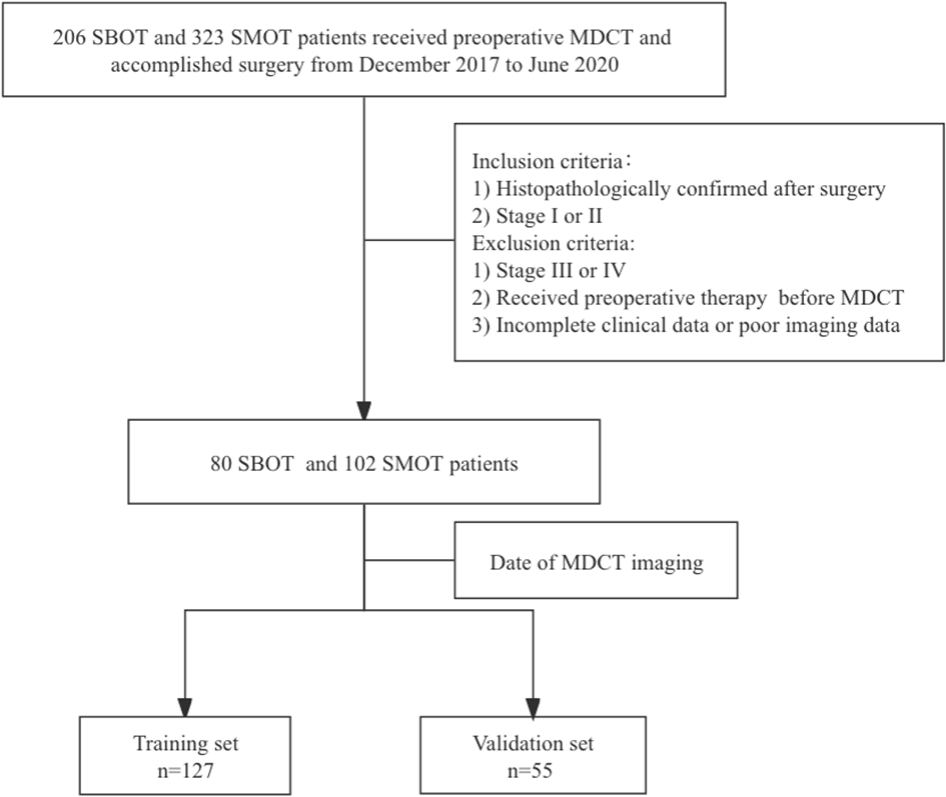
The flowchart of the selection of patients.

This retrospective study was conducted and approved by the institutional review committee, with the requirement of informed consent waived. Eighty SBOT patients (54 stage I and 26 stage II) and 102 SMOT patients (63 stage I and 39 stage II) from December 2017 to June 2020 were enrolled from our hospital information system (HIS) database into this analysis. The inclusion criteria were as follows: 1) histopathologically confirmed SBOTs and SMOTs after surgery and 2) SBOTs and SMOTs diagnosed as stage I or II based on the International Federation of Gynecology and Obstetrics (FIGO) guideline. The exclusion criteria were 1) SBOTs and SMOTs of stage III or IV according to the FIGO guideline, 2) patients that have accepted preoperative therapy (radiotherapy, chemotherapy, or chemoradiotherapy) before MDCT examination, and 3) incomplete clinical data or poor imaging data. The cohort was divided into a training set (127 patients) and a validation set (55 patients) according to the examination date of MDCT, with the total process depicted in **Figure 1**.

### MDCT image acquisition

Pelvic MDCT images from five different CT scanners [AquilionONE (Canon Medical Systems, Otawara, Japan), Discovery CT750 HD (GE Medical Systems, Waukesha, WI, USA), Optima CT670 (GE Medical Systems, Milwaukee WI, USA), iCT 256 (Philips Medical System, Best, Netherlands), SOMATOM Definition Flash (Siemens Medical Systems, Forchheim, Germany)] were obtained with the following parameters: 100–300 mA, 120 kV, pitch of 0.599–0.984, thickness of 1–1.2 mm, and rotation time of 0.42–0.6 s. Iohexol (300 mg iodine/mL) with 85–100 mL volume was administered into the vein using a power injector at the speed of 2.0–3.0 mL/s. Then, the post-contrast CT scans from the arterial phase (AP, 30 s), venous phase (VP, 60 s), and equilibrium phase (EP, 90–120 s) were attained.

### Clinicoradiological risk factors

The clinical characteristics of age, CA125 level (≤50 U/mL; >50 U/mL) (9), and human epididymis protein 4 (HE4) level (≤150 pmol/L; >150 pmol/L) (14) derived from medical records were recorded in the HIS. The imaging was viewed separately and independently by two radiologists in abdominal diagnosis, with 8 and 9 years of experience who were blinded to the clinical variables and pathological diagnosis. The following data of conventional imaging characteristics were recorded: the diameter of the tumor on the axial MDCT image, the diameter of the solid component on the axial MDCT image, location (unilateral or bilateral), texture (predominantly cystic or solid), margins (smooth or irregular), ascites (present or absent), and vascular abnormality (present or absent; vascular abnormality was defined as the presence of no less than one of the following situations: a) chaotic or serpentine course of the vessel, b) microaneurysm, and c) arteriovenous fistula).

### MDCT radiomics analysis

The radiomics analysis methodology was carried out as described in a previous paper (15). In brief, regions of interest (ROIs) were manually segmented with 3D slicer, a free open-source software (https://www.slicer.org/). Afterward, 7 categories and 1,167 radiomics features were extracted with the “radiomics” package of the 3D slicer.

All radiomics features extracted from MDCT of VP were normalized. Significant radiomics features in the training set were selected applying interclass correlation coefficient and least absolute shrinkage and selection operator (LASSO) regression. A radiomics score (Rad-score) of each patient was calculated by means of a linear combination of the chosen features weighted by their coefficients.

### Construction of the differentiation models

Three models were constructed by multivariate logistic regression analysis: the clinicoradiological model by prominent clinical characteristics and conventional imaging characteristics, the radiomics signature model by the selected radiomics features, and the nomogram model by the independent clinicoradiological risk factors combined with the radiomics signature.

### Evaluation and validation of the different models

**Figure 2:**
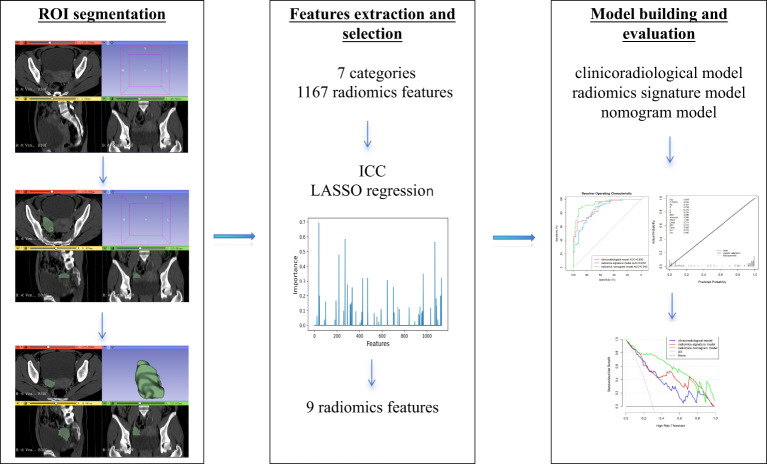
Workflow of features selection and model construction.

The evaluation of calibration of the nomogram model was conducted in the training and validation sets using the Hosmer–Lemeshow goodness-of-fit test. The performance of the three models in diagnosis was evaluated in the training set and validated in the validation set by plotting the receiver operating characteristic (ROC) curve and calculating the area under the ROC curve (AUC) and the multiple comparison of AUC curves using the DeLong test on Bonferroni-adjusted *p*-values. The AUC with 95% confidence interval (CI), sensitivity, and specificity was calculated. Decision curve analysis (DCA) was performed according to net benefits and the corresponding threshold probabilities in the training and validation sets, to assess the clinical application of the nomogram model. Our study workflow diagram is shown in **Figure 2**.

### Statistical analysis

All statistical analyses were carried out on SPSS (version 20, Chicago, IL, USA) and R (https://www.r-project.org/). Statistical significance was defined as *p <*0.05.

## Results

### Patient characteristics

The clinicoradiological characteristics of all patients in the training and validation sets are provided in **Table 1**. SBOTs and SMOTs exhibited significant differences in age, HE4 level, location, texture, margins, vascular abnormalities, and the diameter of the solid component (*p* < 0.05), while there were no significant differences in CA125 level, ascites, and size (*p* > 0.05) in the training set. Age (*p* = 0.001), the diameter of the solid component (*p* = 0.009), and HE4 level (*p* < 0.001) were identified as the independent factors in the clinicoradiological model according to multiple logistic regression analysis. Tumors with older age [odds ratio (OR), 1.11; 95% CI, 1.05–1.18], larger diameter of the solid component (OR, 1.07; 95% CI, 1.02–1.13), and higher HE4 level (OR, 56.94; 95% CI, 7.1–456.49) trended to develop to SMOTs.

**Table 1:** Comparison of the clinicoradiological characteristics of SBOT and SMOT patients.

Clinicoradiological characteristics	Training set			Validation set		
	SBOT (*N*=56)	SMOT (*N*=71)	*p*	SBOT (*N*=24)	SMOT (*N*=31)	*p*
Age (median [range]), years	35 (19–69)	51 (39–79)	<0.01	38.5 (20–67)	53 (41–72)	<0.01
CA125 (≤50 U/mL; >50 U/mL)	20/36	28/43	0.668	11/13	7/24	0.073
HE4 (≤150 pmol/L; >150 pmol/L)	54/2	29/42	<0.01	21/3	14/17	0.003
Location (unilateral/bilateral)	17/39	41/30	0.002	10/14	15/16	0.851
Texture (predominantly cystic/predominantly solid)	47/9	26/45	<0.01	19/5	16/15	0.04
Margins (smooth/irregular)	44/12	36/35	0.002	20/4	18/13	0.051
Ascites (present/absent)	16/40	24/47	0.783	4/20	9/22	0.29
Vascular abnormalities (present/absent)	10/46	26/45	0.022	5/19	16/15	0.024
Size [median (range)], millimeters	72 (32–218)	82 (30–226)	0.272	75.5 (33–133)	81 (43–128)	0.411
Diameter of solid component [median (range)], millimeters	16 (5–80)	42 (5–94)	<0.01	17 (4–96)	38 (9–80)	0.017

SBOT, serous borderline ovarian tumor; SMOT, serous malignant ovarian tumor.

### Radiomics feature selection and model construction

A total of 1,167 radiomics features were extracted from the ROIs, from which 812 with high reliability in the VP were selected for the LASSO to screen out the most contributing features. The radiomics signature model was constructed by the nine eventually chosen radiomics features. The Rad-score was calculated by the formula below:

Rad-score = −34.8136792 + 22.8447829 × Idn + 0.1912723 × ClusterShade − 63.2419151 × RunLengthNonUniformityNormalized − 2.3563718 × Median + 0.9576375 × LowGrayLevelZoneEmphasis + 13.5659235 × GrayLevelNonUniformityNormalized + 28.8492739 × InverseVariance + 11.5428048 × Imc2 − 173.8911646 × Contrast.

### Clinical use of the nomogram model

**Figure 3:**
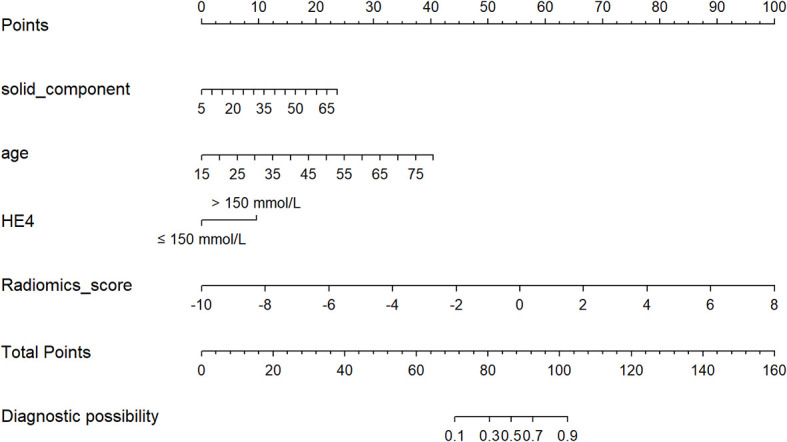
The radiomics nomogram, combining age, the diameter of the solid component, HE4 level, and Rad-score, developed in the training set.

**Figure 4:**
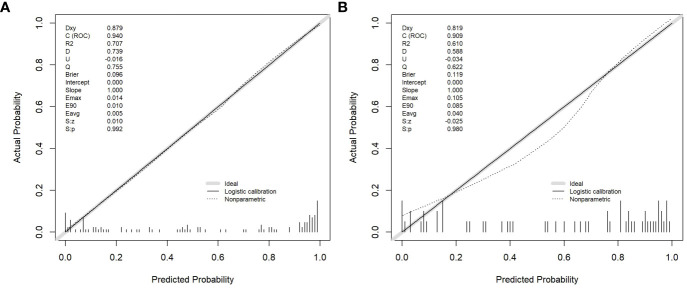
Calibration curves of the radiomics nomogram in the training **(A)** and validation **(B)** sets.

**Figure 5:**
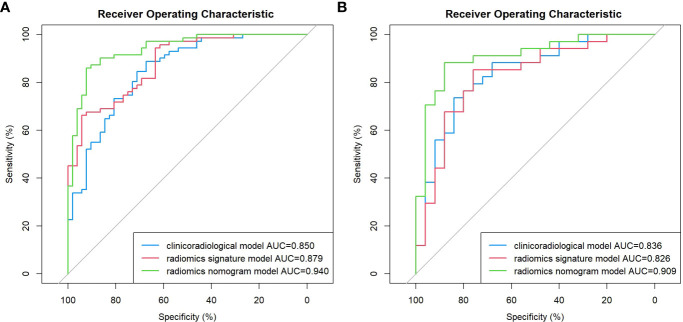
ROC curve of the clinicoradiological model, the radiomics signature model, and the nomogram model in the training **(A)** and validation **(B)** sets.

**Figure 6:**
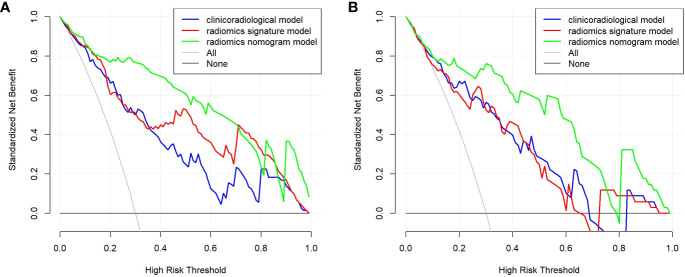
DCA for the clinicoradiological model, the radiomics signature model, and the nomogram model in the training **(A)** and validation **(B)** sets.

Age, the diameter of the solid component, HE4 level, and Rad-score were integrated for the nomogram model construction (**Figure 3**). The calibration curve of the nomogram in **Figure 4** demonstrated considerable calibration in both the training set (*p* = 0.994) and the validation set (*p* = 0.859). The performance of the clinicoradiological model, the radiomics signature model, and the nomogram model in diagnosis is evaluated and summarized in **Table 2**, and the ROC curves are depicted in **Figure 5**. The nomogram model exhibited significantly higher AUC compared with the clinicoradiological model and the radiomics signature model (*p* < 0.001) in the training set, while there was no significant difference in AUC between the nomogram model and the radiomics signature (*p* = 0.123) in the validation set. In the training (*p* = 0.494) and validation sets (*p* = 0.8972), no significant differences in AUC were revealed between the clinicoradiological model and the radiomics signature model. The DCA of the three models (**Figure 6**) demonstrated a more prominent overall net benefit of the nomogram model in differentiating between SBOTs and SMOTs in comparison to the clinicoradiological model and the radiomics signature model.

**Table 2:** Predictive performance of the three models.

	Training set				Validation set				
	AUC (95% CI)	SEN	SPE	*p*	AUC (95% CI)	SEN	SPE	*p*	
1) Clinicoradiological model	0.850 (0.781–0.918)	0.673	0.887		0.836 (0.730–0.943)	0.840	0.735		
2) Radiomics signature model	0.879 (0.822–0.937)	0.942	0.662		0.826 (0.714–0.938)	0.760	0.853		
3) Nomogram model	0.940 (0.899–0.980)	0.923	0.859		0.909 (0.832–0.987)	0.880	0.882		
1 vs. 2				0.494				0.8972	
1 vs. 3				0.00178				0.04379	
2 vs. 3				0.005518				0.123	

AUC, area under the curve; CI, confidence interval; SEN, sensitivity; SPE, specificity.

## Discussion

The biological characteristics, prognoses, and treatments completely vary between SBOTs and SMOTs, implying the crucial role of accurate preoperative differentiation in determining individualized treatment options and improving postoperative quality of life (2). FIGO stage III or IV ovarian tumors more prominently display aggressive characteristics, such as lymphatic and distant metastases, resulting in a tendency to be identified as SMOT (16, 17). Therefore, patients in the early stages were employed as subjects in our study, which holds greater significance in clinical practice.

Age is generally acknowledged as one of the factors that discriminate between SBOTs and SMOTs (18). As indicated by our previous study, the median age of the patients with SMOTs was 10 years older than patients with SBOTs, showing a statistically significant difference (10). In the present study, using multiple logistic regression analysis, age was validated as an independent predictor in accordance with previous studies (18).

In the past two decades, studies on several serum biomarkers for the diagnosis of epithelial ovarian cancer including CA125, carbohydrate antigen 19-9, carcinoembryonic antigen, and HE4 have already been conducted (19, 20). HE4 is a relatively reliable biomarker for ovarian cancer detection, whereas CA125 is commonly used for early ovarian cancer diagnosis in clinical practice (14). In terms of ovarian cancer detection, HE4 exhibits superior diagnostic performance to CA125, while their combination provides higher sensitivity at the expense of lower specificity compared with HE4 alone (21, 22). In the present study, the HE4 level was proven to be an independent predictor in the clinicoradiological model, showing a superior diagnostic performance to the CA125 level in terms of differentiation between early-stage SBOTs and SMOTs.

In routine clinical practice, MDCT is the most commonly used imaging method for preoperative assessment and postoperative surveillance of patients with ovarian tumors. Previous studies have indicated that MDCT and MRI are promising imaging tools for differentiating BOTs from MOTs (23, 24). Due to the focus on variables such as tumor shape, tumor volume, and tumor vascular changes in early studies (6, 17), it is difficult to distinguish BOTs from MOTs depending on imaging information alone, despite the medical imaging methods used to distinguish them, which could result from some morphological imaging findings overlapping between BOTs and MOTs, such as irregularly thickened walls or septa, predominantly solid masses, and vascular abnormalities. Therefore, medical imaging methods must be combined with clinical data to achieve a differential diagnosis. Our previous study demonstrated the validity of the combined analysis of age, vascular abnormalities, and the diameter of the solid components for differentiating between SBOTs and SMOTs (10). Shin et al. (25) found that a more reproducible and accurate differentiation of BOTs from MOTs could be achieved by combining MDCT data and CA125 levels.

With advancements in computer technology, radiomics has emerged as a new research method in recent years. Medical images can be converted into high-dimensional mineable data after the extraction of quantitative image features. Radiomics could contribute to exploring the potential connections related to tumor occurrence and development offering reference for clinicians to make a proper diagnosis before surgery. This is a promising field used in oncology and has been adopted in the differential diagnosis of pulmonary solid nodule (26), renal masses (27), liver tumor (28), and thyroid follicular neoplasms (29) on MDCT images.

In our previous study (15), the MDCT-based radiomics model was efficient in discriminating SBOTs from SMOTs, with AUCs for the radiomics models AP, VP, and EP of 0.8, 0.86, and 0.73, respectively; the AUC was higher for VP. In the present study, we investigated the performance of a nomogram model based on radiomics of the VP for preoperative differentiation between SBOTs and SMOTs.

The nomogram model incorporated a radiomics signature and three clinicoradiological risk factors as follows: age, solid component diameter, and HE4 level. The combined model exerted a more remarkable function compared with the other two models in the training and validation sets. The AUCs of the nomogram model were 0.940 and 0.909 for the training and validation sets, respectively. These findings demonstrated the efficiency of the nomogram model for preoperative differentiation between SBOTs and SMOTs, which is easy to use. The nomogram displayed high consistency and potential clinical usefulness according to the calibration and DCA curves. To the best of our knowledge, for the first time, we constructed a nomogram model with high accuracy and robust discrimination, providing a convenient and non-invasive tool for clinicians.

This study had some limitations. First, it was a single-center scanner study; a multicenter study is warranted to analyze and verify our results in the future. Second, the late stages of SBOMs and STOMs were not included, which may limit the model generalization. Third, there may be an inevitable selection bias in retrospective studies, and prospective and external validation studies are needed. Fourth, the lack of multiple segmentations affected the quality of the study, limiting the reproducibility of the results.

## Conclusions

In summary, a nomogram model combining clinicoradiological risk factors and radiomics features was established in the present study and can be adopted as an individualized preoperative non-invasive tool for distinguishing between early-stage SBOTs and SMOTs.

## Data availability statement

The original contributions presented in the study are included in the article/supplementary material. Further inquiries can be directed to the corresponding author.

## Ethics statement

The studies involving humans were approved by the Ethics Committee of Affiliated Hospital of Qingdao University. The studies were conducted in accordance with the local legislation and institutional requirements. The ethics committee/institutional review board waived the requirement of written informed consent for participation from the participants or the participants’ legal guardians/next of kin because this study was retrospective design, and all data were kept confidential. Written informed consent was not obtained from the individual(s) for the publication of any potentially identifiable images or data included in this article because written informed consent was not required to participate in this study in accordance with the institutional requirements.

## Author contributions

XY: Investigation, Software, Writing – original draft, Writing – review & editing, Methodology. YZ: Methodology, Resources, Writing – review & editing. LW: Methodology, Resources, Writing – review & editing. HJY: Methodology, Resources, Writing – review & editing. JJ: Methodology, Resources, Writing – original draft. HYY: Data curation, Software, Writing – review & editing. SZ: Conceptualization, Methodology, Software, Supervision, Writing – review & editing.
